# Body Aware: Adolescents’ and Young Adults’ Lived Experiences of Body Awareness

**DOI:** 10.5334/pb.1295

**Published:** 2024-08-12

**Authors:** Marbella Pérez-Peña, Jessica Notermans, Jeanne Petit, Katleen Van der Gucht, Pierre Philippot

**Affiliations:** 1 Psychological Sciences Research Institute, University of Louvain, 1348 Louvain-la-Neuve, Belgium; 2 Leuven Mindfulness Centre, University of Leuven, 3000 Leuven, Belgium; 3 Faculty of Psychology and Educational Sciences, University of Leuven, 3000 Leuven, Belgium; 4 Tilburg School of Social and Behavioral Sciences, Tilburg University, Tilburg, Netherlands

**Keywords:** body awareness, interoceptive awareness, adolescence, young adulthood, focus groups

## Abstract

Paying attention to body sensations has been associated with many positive outcomes such as increased subjective well-being, enhanced emotion regulation, and reduced symptom reports. Furthermore, body awareness has an important therapeutic utility in the treatment of various psychological ailments. Despite its importance in mental health, there is very little research on body awareness during adolescence and young adulthood – important developmental periods characterized by bodily changes and the development of one’s relationship to one’s body. Therefore, the present qualitative study sought to explore how body awareness is understood, experienced, and described by adolescents and young adults. Four online focus groups were conducted with young people between the ages of 14 and 24 (*N* = 20). Thematic analyses revealed a multidimensional and highly contextualized understanding and experience of body awareness in this age group. In general, young people reported mainly attending to intense and unpleasant body sensations with a particular attitude (e.g., accepting or avoidant) depending on the type of sensation, leading to a variety of cognitive, emotional, and behavioral reactions to these sensations. These processes were embedded in an underlying schema of beliefs about body awareness and an overarching physical and socio-cultural context. Results further revealed a more nuanced experience and understanding of body awareness in women and in young adults. The present findings can be used as a foundation for the development of body awareness theoretical frameworks and self-report instruments for youth and can aid the generating of hypotheses for future research on body awareness in this age group.

Adolescence and emerging adulthood are crucial periods of development. Numerous bodily changes occur during this period of life such as physical growth, sexual maturation, as well as sleep and circadian regulation changes (Dahl et al., 2018). Furthermore, important habits such as one’s awareness of, relationship with, and image of one’s body begin to form (Laufer, 1996; Lunde & Frisén, 2011). Despite the salient role that the body and awareness of the body play throughout this developmental period, few qualitative studies have explored how adolescents and young adults view, experience, and describe these processes from a subjective point of view.

Interoception can be broadly defined as the sensing, interpretation, and integration of internal bodily signals at conscious and unconscious levels (Khalsa et al., 2018). It is a complex, multi-dimensional construct with a wide array of conceptualizations in the literature (see Mehling et al., 2009). A recent, comprehensive theoretical framework of interoception consists of eight dimensions (see Suksasilp & Garfinkel, 2022 for a thourough presentation of these dimensions). The present article focuses on three of these dimensions that are accessible to awareness and that we consider are of greatest clinical interest: interoceptive attention, self-report and interoceptive beliefs, and attributions of interoceptive sensations. The first dimension, interoceptive attention, refers to people’s tendency to attend to body sensations (Suksasilp & Garfinkel, 2022). For instance, patients with fibromyalgia, chronic fatigue syndrome, and panic disorder report paying significantly more attention to unpleasant body sensations than healthy controls (Bogaerts et al., 2022). Moreover, patients with panic disorder find it particularly difficult to disengage attention from unpleasant body sensations, making this dimension clinically relevant (Bogaerts et al., 2022). The second dimension, self-report and interoceptive beliefs, refers to people’s subjective experiences of their body sensations (Suksasilp & Garfinkel, 2022). This represents a broad dimension measured by self-report questionnaires such as the Multidimensional Assessment of Interoceptive Awareness, which measures a kind of BA characterized by a mindful attentional style (MAIA; W. E. Mehling et al., 2012). Studies using this measure have found that one’s ability to trust one’s body sensations is negatively associated with anxiety and eating disorder symptoms (Brown et al., 2020; W. Mehling, 2016). Finally, the third dimension, attributions of interoceptive sensations refers to people’s appraisal of perceived bodily signals (Suksasilp & Garfinkel, 2022). For example, individuals with panic disorder tend to view body sensations as dangerous and life-threatening (Ehlers, 1993). These three dimensions will be qualitatively explored in the present study and are integrated into the definition of self-reported interoception used in this study: the subjective, phenomenological, and conscious experience of body sensations shaped by complex psychological processes such as attention, attitude, interpretation, and beliefs (Mehling et al., 2011). This definition highlights two key aspects of self-reported interoception: (1) the perceptual and attentional aspects (i.e., consciously perceiving and attending to body sensations), and (2) conscious mental processes that go beyond perception, such as attitude, attributions of body sensations, and reactions to body sensations. The word “body awareness” (BA) will be used for the remainder of the article to refer to self-reported interoception as defined by Mehling et al. (2011).

Researching BA is important because BA plays an important role in psychological well-being and in the treatment of psychological disorders. Various theories emphasize the important link between the different dimensions of interoception and emotional processes (Hölzel et al., 2011; James, 1884), which are key to psychological well-being (Nyklíček et al., 2011). This claim has been supported by research showing that participants who are more aware of their heartbeats, demonstrate increased processing and recognition of emotional stimuli (Pollatos & Schandry, 2008) and report more arousal information when speaking about their emotional experiences (Barrett et al., 2004). Furthermore, adult research has shown that deficits in various dimensions of interoception are associated with many mental health conditions such as anxiety, depression, eating disorders, post-traumatic stress disorder, substance use disorders, and somatic symptom disorders (Khalsa et al., 2018). BA research in youth is mostly quantitative, more predominant in young adult undergraduate samples, and very scarce in adolescent samples. Existing research in youth has replicated the link between BA (particularly self-reported distrust in body sensations) and eating disorder symptoms (Brown et al., 2020), and a positive association has been found between BA and positive body image (Todd et al., 2019). Finally, BA has therapeutic utility in evidence-based psychological interventions such as somatic experiencing (i.e. a form of therapy in which the body’s natural protective reactions and attention to body sensations are used as key therapeutic tools; Brom et al., 2017; Payne et al., 2015), and mindfulness-based interventions, which involve a systematic training in mindfulness meditation practice (De Jong et al., 2016; Fissler et al., 2016; Pérez-Peña et al., 2022). These findings suggest that, in youth, BA may be a protective factor for mental health, whereas atypical BA may be a risk factor for psychopathology.

Studying BA in a population of youth is particularly important because BA may be a key factor in understanding the high rates of psychopathology during this period of life (De Girolamo et al., 2012). Murphy and colleagues (2017) theorize that during adolescence, poor BA skills may lead to risky decision-making and difficulties regulating emotions, which in turn may contribute to increased psychopathology and risky behaviors. However, there is very little research on the developmental trajectory of BA throughout adolescence and young adulthood and on the psychological processes that may be impacted by an atypical BA; hence, more studies are needed to test this theory. Numerous questions remain unanswered. For example, what is the typical development of BA throughout adolescence and emerging adulthood? Do BA skills naturally increase with age and is this negatively linked with psychopathology rates? Do poor BA skills impact decision-making during adolescence?

Before these important questions can be meaningfully explored, there is a need for a BA conceptualization and BA assessment tools adapted to youth. Influential BA theoretical frameworks (Garfinkel et al., 2015; Khalsa et al., 2018; Mehling et al., 2012; Suksasilp & Garfinkel, 2022) have mostly been based on adult research and do not integrate developmental aspects. There has been a call for adopting a developmental neuroscience perspective on BA, as BA may differ with age and have different implications across stages of life (Murphy et al., 2017). This call has been echoed by other authors who highlight the need for a better understanding of the developmental aspects of BA (Khalsa et al., 2018). However, to move the field forward, adequate and valid measures are needed. To our knowledge, there are only two self-report measures evaluating BA that have been validated in a population of youth. The first is the MAIA youth, which measures BA in a multidimensional way, and was adapted and validated in children and adolescents between the ages of 7–17 (Jones et al., 2020). The second one is the Bodily Awareness sub-scale of the Emotion Awareness Questionnaire revised, which conceptualizes BA as maladaptive and focuses on “the physiological aspects of the emotion experience” (Rieffe et al., 2008, p. 757). Both measures have important limitations. The MAIA for youth was initially developed for an adult population based on findings from adult focus groups. Hence, it may not be valid for youth’s experience of BA. The questionnaire was then adapted for youth by changing specific words and phrasing (Jones et al., 2020), but the language may remain overly complex. Regarding the Emotion Awareness Questionnaire revised, it includes a specific dimension of interoception focusing on the bodily symptoms of emotions and hence is not appropriate for a broader assessment of adaptive BA. In short, there is a need for BA theoretical frameworks in youth and BA measures grounded in these frameworks for research in the field to progress.

The purpose of the present study is to gain insights into how BA is subjectively understood, experienced, and described by adolescents and young adults in order to inform BA theoretical frameworks and self-report measures tailored to youth. The nature of this research is exploratory and hypothesis-generating rather than confirmatory or hypothesis-driven. To reach the study’s aim, a series of focus groups (FGs) with young people between the ages of 14 and 24 were conducted. The age range of 14–24 was chosen because we wanted to include participants with sufficiently established self-reflection capacities. The capacities of self-reflection, meta-cognition, and self-evaluation become increasingly established from mid-adolescence onward with the maturation of the frontal lobes (Steinberg, 2005). A qualitative approach was chosen because of its potential for identifying important domains of interest in the topic of BA in youth (Carey & Asbury, 2016), which is under-explored and poorly understood (Jones et al., 2020; Murphy et al., 2017). Furthermore, BA research in youth has focused on quantitative methods using measures and definitions of BA borrowed from research with adults (Emanuelsen et al., 2015; Todd et al., 2019), which may not be directly transferable to this age group. To our knowledge, existing qualitative studies that explicitly focus on the subjective experience of BA have been performed on adult populations (e.g., Mehling et al., 2011). Existing qualitative studies with youth include BA as a secondary measure, but not as the main focus of their research question (e.g., Bugge et al., 2012). Therefore, the present study will explicitly focus on the subjective experience of BA in a sample of young people. The aim is not to generalize the findings, but to provide rich, in-depth, and detailed data on the experience of BA in a sample of youth.

## Methods

### Participant recruitment

Recruitment began on February 7th, 2020 and ended on March 23rd, 2020. Adolescents and young adults were recruited via various means. The research team sent a recruitment email to five youth movements (i.e., organizations for leisure time activities such as the scouts) and two secondary schools in Brussels and vicinity, to acquaintances of the research team, as well as to students in the Faculty of Psychology and Educational Sciences of the University of Louvain. A recruitment post was also shared on social media platforms (i.e., Facebook). The invitation included a brief description of the objectives of the study, its modalities, and the compensation. Participants were invited to contact the researcher if they were interested in participating. The inclusion criteria were: (1) being between the ages of 14 and 24, (2) speaking French fluently, and (3) having access to a device with internet connection, as the FGs were conducted online due to the Covid-19 pandemic. Interested participants were then e-mailed information sheets explaining the details of the study (minors were emailed two information sheets, one for the teenager and one for the parents, and a parental consent form to be signed and sent before the FG), as well as a Doodle link to schedule the date and time of the group. Once the date and time were confirmed, a final e-mail was sent containing practical details and the following documents: a list of the five questions that would be asked during the virtual FG, the informed consent form to be read prior to the FG, and an instruction sheet explaining how to use the virtual platform Adobe Connect (Adobe Connect, 2021). All participants below the age of 18 sent us signed, written parental consent forms, and all participants electronically signed written informed consent forms prior to the start of the virtual FG, including the permission to audio-record and transcribe the discussions, as well as to publish the results. The study was conducted according to the guidelines of the Declaration of Helsinki and approved by the Ethical Committee of the first author’s university (Projet2020–07; approved on January 29th, 2020).

### Data collection

A total of 20 young people between the ages of 14 and 24 agreed to participate in the online FGs. All participants who were recruited participated in the study. FGs were chosen for data collection in order to explore youth’s experiences, attitudes, and opinions on BA in a rich way (Ravitch & Carl, 2016). Due to their interactive nature (e.g., sharing a diversity of viewpoints and building on each other’s ideas), FGs may provide richer data than individual interviews (Guest, Namey, Taylor, et al., 2017). We originally planned to conduct the FGs in person but the Covid-19 lockdown obliged us to conduct online FGs, which have both limitations and advantages (see Tuttas, 2015). The groups were divided by age in order to create a more comfortable and less intimidating environment in which participants could express themselves freely. Moreover, we divided participants by self-reported gender (i.e., including the options male, female, and other) to foster an open and authentic expression which may be hindered in mixed-gender groups (Millward, 1995). Participants were divided into one of these two groups based on their self-reported gender, with one exception. One participant identified as transgender and was assigned to the group of their sex assigned at birth with the participant’s consent. We conducted a total of 4 FGs (2 groups per developmental stage and 2 groups per gender) with 4 to 7 participants per group. Group number was based on research showing that 2 to 3 FGs is enough to reveal 80% of discoverable themes (Guest, Namey, & McKenna, 2017), and group size was based on literature showing that 4 to 10 participants is enough, as smaller groups provide richer data (Carey & Asbury, 2016; Millward, 1995; Sweet, 2001).

Participants in FG1 were female adolescents (*N* = 4; mean age = 15.75; *SD* = 0.5; age range: 15–16) and were secondary school students in 9th (*n* = 1), 10th (*n* = 1), and 11th grades (*n* = 2). Participants in FG2 were female young adults (*N* = 7; mean age = 22.29, *SD* = 1.98; age range: 19–24), and were university students at the bachelor (*n* = 2) or master level (*n =* 5), studying human sciences (*n* = 6) and audiovisual studies (*n* = 1). Participants in FG3 were male adolescents (*N* = 5; mean age = 15.6, *SD* = 0.55; age range: 15–16), who were mostly secondary school students in 9th (*n* = 2) and 11th grade (*n* = 2). Finally, participants in FG4 were male young adults (*N* = 4; mean age = 20.25, *SD* = 1.71; age range: 18–22), and were all university students at the bachelor level studying human sciences (*n* = 3) or biomedical sciences (*n* = 1). All participants’ mother tongue was French, and most participants were of Belgian nationality (except for one participant in FG2 who was of French nationality). See Supplementary Materials for more details on participants’ demographic characteristics.

The online FGs were conducted between March and May 2020 on the web conference platform Adobe Connect (Adobe Connect, 2021), which was selected according to the eight criteria outlined by Tuttas (2015): the platform should (1) allow for meetings with up to 10 participants, (2) allow for real-time audio and video, (3) permit audiovisual recordings of the discussion that only the host would be able to record, (4) make audiovisual recordings accessible to the host only, (5) be easy to use for participants, (6) make it easy for participants to join the meeting, (7) require no purchasing or installation from the part of participants, and (8) only allow invitees to access the meeting.

The duration of the online FGs was one hour and a half. The groups were co-facilitated by three members of the research team (MP, JN, JP). Two of the co-facilitators took turns leading the discussion and taking notes on verbal and non-verbal communication. The third co-facilitator oversaw technical aspects (e.g., helping participants if they encountered technical issues, recording the session, copy pasting the questions in the virtual discussion board, timekeeping). Regarding positionality, all three co-facilitators were cisgender women of Latin American or Western European origins, conducting psychological research at the master or doctoral level with a focus on the topics of body awareness, mindfulness, and youth. At the beginning of the session, participants were asked to sign the informed consent form electronically and to fill in a short demographic survey regarding gender, age, nationality, mother tongue, the year and orientation of their educational studies, and whether they would like to receive the results of the study. At the end of the FG, participants were asked to fill in a short survey asking them to evaluate their experience of the virtual FG. Young adult participants received 8 euros for their participation and adolescent participants received a store voucher of 10 euros for their participation.

The multidimensional conceptualization stated in the introduction was used to draft the questions. Hence, questions included the perceptual aspects of BA, as well as the conscious mental processes beyond perception, such as attitudes, reactions, beliefs, and interpretations of body sensations. The following questions were asked:

1. Do you remember a time when you felt a sensation in your body (e.g., stomach rumbling, eyes stinging) due to a specific state (e.g., when you felt a particular emotion or were hungry, thirsty, or tired)? What did you feel? Did you notice what you were feeling while you were feeling it or afterwards?
2. During the day, what kind of sensations do you pay attention to in your body?
3. When you notice a sensation in your body during a particular state (e.g., because you felt an emotion, or perhaps you felt tired or hungry), what do you do? Please give a concrete example from your daily life.
4. Do you think that paying attention to body sensations is helpful, not helpful, or not important to you? Could you give concrete examples of times when it was helpful and/or times when it was not helpful/or times when it was bad to pay attention to your body sensations?
5. In your experience, is there a connection between noticing sensations in your body and noticing the state you are in at that moment? Why or why not? Can you give examples from your daily life?

### Data analysis and interpretation

All FG sessions were audiovisually recorded. Immediately after the FG, the co-facilitators (MP, JN, JP) did a debriefing to go over the main observations. Soon after this, the core qualitative team (MP, JN, JP) conducted a preliminary analysis that involved thoroughly reviewing the notes taken during the FG session, listening to the recordings, and noting down important observations. Next, the team conducted verbatim transcriptions of all FG sessions and checked all transcripts for accuracy. The data was then analyzed using a team-based thematic analytical approach derived from the approaches described in Braun and Clarke (2006) and Ravitch and Carl (2016). A thematic analytical approach was chosen because the goal was to identify and analyze patterns in the data. Thematic analyses were conducted separately for young adult and adolescent FGs, leading to two sets of themes. The approach involved: (1) familiarizing ourselves with the data by reading through it multiple times, highlighting text that stood out, and jotting down initial impressions, (2) generating codes (i.e., the most basic piece of information that can be assessed meaningfully; Boyatzis, 1998) (3) searching for themes based on the different codes, (4) reviewing the themes, (5) defining and naming the themes, and (6) writing up the results. These steps were followed by the members of the core qualitative team. NVivo was used for steps one through five (QSR International Pty Ltd, 2020). Regular meetings were held to come to a consensus on the codes and themes, and to further discuss the themes with the other team members. Intercoder reliability is not reported because its value is questioned in thematic analysis, a purely subjective approach (Vaismoradi et al., 2013).

## Results

The resulting themes of our thematic analyses can be found in Table 1. Results are discussed for adolescent and young adult groups separately and include quotes reflecting the essence of each theme. In order to be concise, not all relevant quotes were included under each theme, but interested readers can refer to the Supplementary Materials to find additional quotes.

**Table 1:** Overview of themes and sub-themes of adolescent and young adult focus groups.


ADOLESCENTS	YOUNG ADULTS

1. Attention Attention grabbers (intensity, novelty^W^, problematic^W^, and unpleasantness) Temporality of attention	1. Attention Attention grabbers Temporality of attention Types of attention (voluntary and non-voluntary)

2. Awareness of body sensations Types of sensations (emotions, muscular, and physiological needs) Contextual influence Lack of awareness	2. Body sensations Types (emotion-related, physical, and undefined) Impermanent^M^

3. Reactions to body sensations Hedonic (feel pleasure and avoid pain) Accepting Listening Instrumental	3. Processes Labeling body sensations Interpretation Attitudes (hypervigilance-driven, mindful, and trusting^W^)

4. Beliefs about body awareness Adaptivity Consequences of not listening Good balance Link between states and sensations	4. Factors influencing body awareness Activities Sociocultural context Past experiences^W^ Physio-temporal context (e.g., time of day) Mood or state

	5. Reactions to body sensations Reactions to physical sensations Reactions to emotion-related sensations (accepting, being submerged, analyzing, avoiding, approaching, and using body sensations to regulate) Reactions to undefined sensations

	6. Mind-body connection Mind-body distinction^W^ Sensations, states, and mind

	7. (Mal) adaptivity of body awareness Adaptive Maladaptive


*Note*: ^W^ = sub-themes present only in the women’s discourse. ^M^ = sub-themes present only in the men’s discourse.

### Adolescent results

#### Attention

With regards to attention, adolescents spoke of the characteristics of body sensations that immediately capture their attention, and the exact moment in which they notice these sensations, during or after they are experienced.

##### Attention grabbers

Adolescents, in both the female (FG1) and male FGs (FG3), reported a general lack of attention to body sensations in their daily lives unless sensations were intense, novel, unpleasant, or problematic. When asked what kind of body sensations they paid attention to throughout the day, most adolescents spoke of intense body sensations such as sensations associated with strong emotions or sensations linked with accumulated fatigue.

*If I’m running and um in that moment I pay attention because during a sprint we feel tired suddenly and then really good and since the sensation is intense I pay attention but if not it’s really rare. (FG3)*

Female adolescents also spoke of paying attention to body sensations that were out of the ordinary or not part of their everyday life, such as becoming very angry or experiencing a big stress. However, some female adolescents pointed out paying attention to everyday body sensations that were considered problematic such as sensations of tiredness, because even though these sensations were not novel or unusual, they prevented them from accomplishing tasks in their daily life (e.g., falling asleep at night, performing well during a piano audition, starting their day).

Finally, the negative valence or unpleasantness of body sensations was also an important characteristic that captured adolescents’ attention in an instant and automatic way. Attention to these sensations was motivated by a desire to fix the unpleasant sensations or make them go away.

*I personally pay attention more quickly to unpleasant sensations, except when I really want to enjoy a moment…then I pay attention to pleasant sensations but it’s not automatic, but for unpleasant sensations it’s really automatic because it’s something that bothers me or something and so I try to pay attention to fix it, in any case it’s more automatic. (FG1)*

##### Temporality of attention

This refers to the moments in which adolescents pay attention to body sensations, when perceiving them and/or afterwards. When body sensations were intense, novel, problematic or unpleasant, adolescents reported paying automatic and immediate attention to their direct bodily experience. However, when body sensations were milder or when adolescents were engaged in other activities, body sensations were usually not attended to in the moment but afterwards (when their intensity increased or otherwise changed).

*So I feel sensations during when they are strong emotions but when they’re small for example a mild tiredness well I feel them as they accumulate and so I don’t feel them necessarily in the moment itself but when I’m for example extremely tired or angry, well I feel it more. (FG1)*

#### Awareness of body sensations

Awareness of body sensations refers to the aspects of awareness that adolescents alluded to in the FGs, mainly the content of awareness (i.e., types of body sensations), context, and lack of awareness of the body.

##### Types of sensations

When adolescents spoke of the different body sensations they attended to in their everyday lives, they spoke of three categories of sensations. First, most adolescents reported being aware of body sensations related to emotions, mainly body sensations related to stress, anxiety, and anger. A few adolescents spoke of body sensations related to sadness and joy. Concerning anxiety, one participant in FG1 expressed: *“When I start to find it difficult to breathe or I have a weight on my lungs I know I’m anxious.”* Second, various adolescents also identified muscular body sensations linked to posture and muscles particularly in the context of doing sports: *“When you do sports, well, the body sends signals that you are suffering… For example, your muscles are aching, you are tired, you are out of breath, your heart is beating faster, uh, sweating and all that.” (FG3)* Lastly, adolescents spoke of a category of body sensations related to the body’s physiological needs such as the need for food or rest expressed as sensations of hunger and fatigue respectively.

##### Contextual influence

Adolescents spoke of the contexts, times of day, and activities in which they paid attention to body sensations. Most mentioned paying attention to body sensations when they were doing some form of physical activity (e.g., at the gym, hiking, sprinting, dancing), during stressful moments (e.g., exams), at specific moments in the day (i.e., when waking up and going to sleep), in social contexts (e.g., when with family), and when alone.

*Otherwise it’s true that in the evening, for example, when I lie down in bed, I pay attention, especially when I can’t sleep, I say to myself: What do I feel here? What do I feel here? And except for the moments when it’s really a moment alone or I don’t have to do anything but otherwise as soon as I’m doing something else, I don’t think about the sensations in my body. (FG1)*

##### Lack of awareness

Adolescents reported a lack of awareness of body sensations in certain contexts. Most adolescent boys reported a general lack of attention to their body sensations except when there was a physiological need or pain in the body.

Adolescent girls, on the other hand, reported specific moments when they were not aware of their body sensations; mainly when completing tasks or when at school.

*Okay, um, but it’s true that the time when I’m least aware of the sensations in my body is as soon as I’m busy because as soon as I have to think about something else, about work, about doing sports or whatever, well, unless it’s a really big pain, I’m not going to feel it. (FG1)*

#### Reactions to body sensations

Adolescents spoke of four main kinds of behavioral and emotional reactions to their body sensations: (1) hedonic, (2) accepting, (3) listening, and (4) instrumental.

##### Hedonic

Most adolescents expressed a hedonic way of relating to their body sensations. They actively sought pleasant sensations and tried to make them last, enjoy them, repeat them, and remember them. With regards to unpleasant sensations, they reported avoiding and rejecting these sensations and seeking ways to make them go away: *“When I have a sad sensation, I don’t know, something like that, I try to think of something else… Mmhmh, something nice, find an activity that makes me forget a bit.” (FG3)*

##### Accepting

Some adolescents reported reacting to body sensations with an attitude of acceptance; particularly when it came to body sensations associated with intense unpleasant and pleasant emotions. Acceptance is defined as a way of relating to experiences with present moment awareness, openness (i.e., giving space to the experience without changing it) and self-compassion (i.e., acceptance of one’s limits and imperfections; Philippot, 2011). One participant describes this attitude very clearly.

*I think you have to take care of yourself and ask for help if you need it, but sometimes you have to tell yourself that it’s normal…There are certain periods when you are a little bit, well I’m a little bit fragile and I know that I can cry for a yes or a no and two minutes later I say to myself, well shit, I shouldn’t have cried, but it’s not a big deal…I think we have to be careful but we also have to tell ourselves that our body is not perfect and that sometimes there are things that don’t work and we can’t explain everything. (FG1)*

##### Listening

Various adolescents reported reacting to body sensations by simply listening to them. This refers to paying attention to sensations and acting in accordance with what they communicate. Such a reaction is mainly evoked in relation to physiological needs (e.g., eating when hungry, drinking when thirsty, sleeping when tired) and to sensations of pain (e.g., changing positions or stopping an activity when the body gives signals of pain). For example, a participant in FG3 expressed: *“When I’m tired, I don’t feel like doing anything and my eyes sting, so I go take a nap.”*

##### Instrumental

Several adolescents stated having an instrumental response to bodily sensations, handling them in ways that promote the accomplishment of specific tasks or goals. Performance and evolution are goals that were particularly present in the discourse of some boys. They explained that in sporting or emotional contexts, it is sometimes necessary to divert attention from bodily sensations in order to evolve.

Some adolescents also discussed the usefulness of bodily sensations, the aim being to listen only to the sensations they consider useful and to repress or transform those they consider unnecessary, or which are not advantageous for the accomplishment of an objective, for example the good progress of an oral presentation:

*It’s true that there are times when uh me too I repress the sensations I feel, so when it’s also not really useful (…) I have to present something in front of the class well then I start to have a lump in my stomach and it’s really not the time because I have to present my thing and it’s graded and all that and so at that time I I repress a little bit what I feel inside to try to manage my stress and still present something good” (FG1)*

#### Beliefs about BA

Finally, adolescents spoke about their beliefs regarding the utility and dangers of BA as well as the link between body sensations and emotional and physical states. Four main types of beliefs were present in the adolescent discourse, each of which is described below.

##### Adaptivity

Various adolescents expressed the belief that BA is adaptive because it allows them to become aware of their physical and emotional state, their needs, and their limits. It also helps them gain a better understanding of themselves.

*Participant: I don’t know if it [BA] can make us feel better, but it can help us understand ourselves, which I think is always helpful*.

*Facilitator: What do you mean by understanding ourselves?*

*Participant: Well, for example, for the emotions, by noticing when there was an emotion and when there wasn’t, we can establish causes and consequences and then know what puts us in this emotion and what puts us in another emotion and then know how we can choose the emotions we feel or things like that… (FG3)*

##### Consequences of not listening

Various adolescents expressed their belief that not listening to body sensations is harmful because when one does not listen to body sensations or when one suppresses them, these sensations become more intense and problematic, and may even lead to physical symptoms or an explosive reaction.

*I think it’s very important to pay attention to them [emotion-related sensations] quickly enough, not always at the same time, but to pay attention to them and not to repress them, because things can really get out of hand if we wait too long… (FG1)*

##### Good balance

Although adolescents reported believing that BA is generally adaptive, they added that it is only adaptive under certain conditions. According to most adolescents, paying a balanced attention to body sensations is important but paying too much attention (i.e., abstract rumination, obsession, being overwhelmed by them) should be avoided.

*I think that in most cases it’s [BA] useful but not in all cases like in anger for example, if you think about it too much it can make the situation worse, so it’s a bit case by case that, sometimes it’s useful to think about it and sometimes not…it’s good to know that I’m angry, but then to be able to move on. (FG3)*

##### Link between states and sensations

Most adolescents reported believing there is a link between body sensations and physical or emotional states. They spoke of body sensations as signals of particular states (e.g., hunger, emotions). They reported that most of the time, body sensations and states are coherently connected (i.e., neutral body sensations indicate a neutral emotional state) whereas other times there is a perceived incoherence (i.e., body sensations signal a state that is incoherent with what one expects to feel or experience).

*Well, I would say that the body is finally the mirror of our sensations because all the emotions that we feel in us will always be translated in our body by… a discomfort or even a pain or a stupid sensation, a tingling or something like that, it’s really a mirror and even if sometimes we don’t realize that both are there I think that very often both are there. (FG1)*

### Young adult results

#### Attention

Like adolescents, female (FG2) and male young adults (FG4) also spoke about different aspects of attention to body sensations, mainly the characteristics of body sensations that most capture their attention, the moment in which body sensations are noticed, and whether sensations are attended to automatically or voluntarily.

##### Attention grabbers

Like the adolescent discourse, young adults reported noticing intense and/or problematic body sensations very quickly. Several young adults reported not paying voluntary attention to their body sensations throughout the day. However, when intense (e.g., sensations of being in love, enthusiasm of going to a party) or troublesome sensations (e.g., sensations that distract them from the task they are doing) arose, they paid attention in order to understand them or take some action: *“When it’s really strong sensations in the body and it’s really a problem for me, I know that I’m going to really listen and then I’m going to stop doing whatever it is that’s causing me that sensation.” (FG2)*

##### Temporality of attention

Similar to adolescents, young adults described paying attention to body sensations at different moments in time. Some young adults reported noticing body sensations as they occurred in the present moment and were also capable of immediately linking the sensation to a physical or emotional state. Others reported not noticing body sensations in the moment in which they occurred, but afterwards when they were trying to make sense of their prior experience. Yet others noted not paying voluntary attention to body sensations in the moment unless the sensations were very strong, which is in line with the Attention Grabbers sub-theme described above.

*…well if it’s a negative sensation in any case, maybe the positive ones, I don’t get them directly but it’s afterwards when I come back maybe in a more normal atmosphere, we’ll say like if I come back to my family after a night out with my friends, well I’m going to feel… well I’m going to start to understand the kinds of sensations that I had with my friends. (FG4)*

##### Types of attention

Two distinct types of attention were present in the young adult discourse: automatic attention (i.e., paying attention to body sensations automatically) and voluntary attention (i.e., voluntarily paying attention to body sensations). There were many examples of automatic attention, particularly in the male group, where we observed a continuum between ignoring body sensations throughout the day and being unable to disengage attention from body sensations.

*Yes, I find it difficult to get my mind off the sensations, especially the stressful ones, that my body imposes on me, for example, I recently suffered from tinnitus, during which time I thought about it all day… Generally, I have difficulty detaching my attention from my body sensations if I am not engaged in a particular task. (FG4)*

Young adults also reported paying deliberate attention to body sensations throughout the day. This was most prevalent in the female group. Participants reported voluntarily paying attention to sensations during physical activity, when experiencing certain emotions, or at random moments in the day to simply come back to the present moment.

*…several times a day, even I would say yeah several times a day anyway because almost every hour in my opinion there is at least one time when I say to myself uh where am I? Uh what is it? I mean I tell myself come back to your body or I immerse myself in the present moment through my senses or I’m trying to cut off the mind. (FG2)*

#### Body sensations

During the FGs, young adults spoke of the content of their BA. They spoke of the different types of body sensations they observed in their direct experience as well as the impermanent quality of body sensations.

##### Types

Young adults spoke about the different types of body sensations they were aware of. They identified three main categories of body sensations: (1) physical sensations (i.e., body sensations related to physiological needs like hunger; physical sensations such as pain, muscular tension or release; and body sensations coming from the 5 senses), (2) emotion-related sensations (i.e., body sensations experienced during emotional states), and (3) undefined sensations (i.e., body sensations that do not fit under the previous two categories and whose meaning or source is difficult to understand, such as an undiagnosed medical condition).

*Yes, for me there are different uh there are many different emotions or sensations that we can feel, for example there is the sensation of hunger, tiredness uh anger yes, it’s as if it was linked to emotions in fact, for me the sensations can be intimately linked to emotions. (FG4)*

##### Impermanent

Young adults (particularly men) expressed a clear awareness of the changing nature of body sensations. Some examples participants mentioned include: pressure in the body being eased by breathing, contraction turning into relaxation, and complex emotions disappearing with time.

*Participant: I have the impression that the sensations are evolving*.

*Facilitator: What do you mean by the sensations evolve?*

*Participant: That this pressure is evacuated by my breath… That while I am in the same room as the person who mobilizes anger in me I feel a certain pressure… and when I leave this room, I feel my body relax… The parts that I felt contracted… are relaxed and I feel that it is by breathing out that the pressure decreases. (FG4)*

#### Processes

The young adult discourse revealed three distinct psychological processes involved in BA, mainly the labeling of, cognitive interpretation of, and attitude towards body sensations.

##### Labeling body sensations

Several young adults reported that when they experience a body sensation, they often label it as physical, emotion-related, or undefined and based on the label, they choose how to best deal with it.

*Personally, what I do is that when I feel a sensation in the body, I categorize it in itself, I make the difference between a sensation which is precisely provoked by an emotion and therefore there is the mental work to be done and thus to see a little bit what there is to do, the sensation which is caused by a more normal state like hunger, fatigue, thirst which can be satisfied, well-regulated in a rather direct way, and then there are sensations which are precisely a little less normal but which are not going to be linked to something emotional, which require a little more analysis… And so it is on the basis of this categorization finally that there is action or inaction which is chosen… (FG2)*

##### Interpretation

On top of categorizing body sensations, young adults also spoke about their cognitive interpretation of body sensations (i.e., the meaning they ascribed to them). Some interpretations included: sensations as symptoms, sensations as a reminder of one’s sex assigned at birth (in the case of a transgender participant), and sensations as indicators of things one does not like in life. For example, a participant in FG4 expressed: *“If I realize that this sensation is a symptom, I’ll tend to tell myself ‘It’s just a message from my body’ and I’ll be able to ignore it more easily.”* Furthermore, a couple of participants highlighted that it’s not the body sensations themselves that can be problematic but one’s interpretation of them.

##### Attitudes

Young adults spoke of different attitudinal stances toward body sensations. Three main attitudes emerged from the discourse: (1) hypervigilance-driven, (2) mindful, and (3) trusting.

A hypervigilance-driven attitude was characterized by an abstract ruminative focus on unpleasant body sensations. Young adults used words such as “a negative spiral”, “an obsession”, and “an over-consciousness” when describing this attitude.

*Sometimes if something happens in our body, we will want to analyze it at all costs and so on and we will want to know exactly what it is and this can turn into an obsession or we can misinterpret it or something like that and I think that it can be harmful because it makes us ruminate…(FG2)*

A few young adults also spoke of instances when they mindfully approached body sensations. This kind of attitude was characterized by focusing attention on present-moment sensations without trying to change them. Of the three attitudes cited in this theme, this was the least commonly mentioned amongst young adults.

*…almost every hour in my opinion there is at least one time when I say to myself uh where am I? Uh what is it? You come back to your body or I come back to the present moment through my senses or I try to cut off the mind… (FG2)*

Finally, several young adult women spoke of a third attitude towards body sensations: trusting. This attitude was characterized by the belief that body sensations were often truer indicators of one’s needs, wants, and dislikes than thoughts. From the viewpoint of this trusting attitude, body sensations were seen as a trustworthy internal compass that must be listened to.

*I find that in fact both in the example to know where we are in our cycle and to know well what corresponds to us or not uh staying in the thoughts, in the mind it doesn’t give a real indicator in my opinion, so it is only the, well through the body that we have true information. (FG2)*

#### Factors influencing BA

This theme refers to the contextual, individual, and social aspects influencing young adults’ awareness of body sensations (i.e., making them more or less aware). The young adult discourse revealed five main factors influencing their BA, each of which is described below.

##### Activities

Various young adults reported an increased and deliberate awareness of body sensations during particular activities such as: drinking or smoking a joint, doing sports, experiencing emotions, and before and after eating. For example, a participant in FG4 expressed: *“On the other hand, when I drink or smoke a joint, I will pay attention on purpose to the sensations that appear, it will not be automatic anymore.”*

##### Sociocultural context

Some young adults also spoke of the effect of social and cultural aspects on their BA. For instance, regarding social context, one young adult reported paying less attention to body sensations when with friends and more attention when with family. With regards to cultural context, the young adult women discourse showed that the cultural ideals of beauty made some of them hyper-aware of their body (e.g., size of breasts and hips, weight, body size). From this perspective, the young adult understanding of BA expands from awareness of internal body sensations to awareness of external body image as well.

*At the beginning of adolescence if we take for example the fact that we begin to put on… if we have very strong awareness of our body and thus we are aware of the breasts which develop or of certain shapes which appear or of certain parts which put on a little weight and we have a strong awareness of it when around us finally there are other girls who can’t put on weight or that there is a model of beauty which is not validated, all of a sudden this bodily awareness, we are so aware of our body that it becomes a hyper-awareness. (FG2)*

##### Past experiences

Young adults (in the women’s group only) spoke about past experiences that augmented their BA. Participants said that after experiencing medical problems (i.e., lung and back problems) they became more attentive of their bodily signals. Other participants reported that their level of BA increased greatly after receiving specific trainings or therapies (i.e., hypnotherapy and a particular training with horses).

*I had a pneumothorax…it’s a small problem in the lung and it’s probably the physical sensation that I felt the most… This event really increased the attention I gave to my own body, not only what I was feeling inside, because before that it’s true that I was like everyone else only paying attention at the external physical level or to sensations of touch but that really… I started to pay attention to the slightest little pain, to the slightest little pull, the slightest, the slightest sensation really that there was. (FG2)*

##### Physio-temporal context

This sub-theme refers to the influence of physical context and time of day on young adults’ BA. Young adults reported being more attentive of their body sensations when in front of a mirror, in the shower, or in bed. For example, one participant in FG4 expressed: *“When I shower in the morning, I feel like I pay close attention to the messages my body sends me.”* With regards, to time of day, most young adults reported an increased BA in the morning when waking up or in the evening before sleeping. One participant mentioned deliberately paying attention to body sensations before sleeping in order to fight insomnia.

##### Mood or state

A few young adults reported that their mood or state influenced their level of BA. For instance, sensations became more intense when in a state of fatigue leading to increased BA. Furthermore, another participant mentioned that one’s mental state can define whether BA is helpful or not.

*If, well if I’m tired basically uh it’s, the sensations are going to be stronger in fact and uh well the negative situations I’m going to be even more sensitive to them and if they are positive it won’t change. (FG4)*

#### Reactions to body sensations

In line with categories of body sensations outlined above, young adults reported reacting to body sensations in a specific way depending on the type of sensation they experienced.

##### Reactions to physical sensations

This sub-theme refers to how young adults react to body sensations related to physiological needs or physical pain. Similar to adolescents, young adults generally approached physical sensations by using a method of problem-solving. More specifically, if a particular body sensation indicated a physical need (e.g., hunger), then they met the need (e.g., eat); if a sensation indicated that something was wrong (e.g., pain), then they found a solution (e.g., stretching, massaging). For physical sensations that could not be resolved so easily (e.g., menstruation-related sensations), the approach was to simply do nothing and go on with one’s day despite the discomfort.

*…when I have my period for example, because it’s true that it’s a state where I’m totally conscious of what’s going on but I don’t do anything about it, I don’t know how to say it, I try to go on with my life with the inconveniences (laughs). I think it’s really a particular moment where I try to get over it and to uh, because there’s not much you can do about it (unintelligible). (FG2)*

##### Reactions to emotion-related sensations

Regarding reactions to body sensations linked to emotions, the young adult discourse was very rich. In contrast to physical sensations that could be easily “fixed” or “solved”, young adults reported that handling emotion-related sensations was less straightforward. They hence reported a diversity of reactions to emotion-related sensations. The problem-solving approach was still present as various young adults talked about analyzing their emotion-related sensations (i.e., thinking about their source) in order to act and attenuate the sensations. For example, a participant in FG4 expressed: *“…when it’s an angry feeling and so on, trying to think about it, to see why it’s there, how, what can we do to solve it. And if it can help us to calm down this feeling…”*

When the problem-solving approach did not work (e.g., when feelings were intense), young adults used other methods such as simply accepting the emotion-related sensations (i.e., letting them follow their course and not doing anything to change them): *“…a grief that you can’t really uh do anything about, it’s more interesting to get over it.” (FG4)* Another method used was approaching the sensations (i.e., feeling what you feel).

Several young adults reported having difficulties dealing with unpleasant sensations linked to negative emotions. In these cases, some participants reported feeling submerged or taken over by the sensations:

*In general, what blocks me more physically speaking is especially when it’s emotions, often sadness or things like that, and there, well, that blocks me and, uh, I don’t know how to do anything when I’m sad, or let’s say I’m dumped, or something like that, well, I wouldn’t be able to eat, or things like that, and, uh, and that’s where I feel it much more in relation to emotions. (FG2)*

Other participants reported trying to avoid feeling these sensations altogether: *“I find that there are more sentimental sensations or even bodily sensations…which are better to put blinders on because sometimes they can do more harm than anything else…” (FG4)*

Finally, some participants talked about reacting to emotion-related sensations by using the body to regulate the intensity of their emotional experience.

*I think that for example if I am angry well I will go to well be alone somewhere, to calm this anger all alone, I don’t know I will try to run or to make a physical movement to try to release a little this anger which is in me by putting energy into what I am doing with my body, to expend this anger… (FG4)*

##### Reactions to undefined sensations

A few young adults spoke about their reactions to undefined sensations (see above for definition). Once again, different young adults reported different reactions. Some engaged in negative thinking and their mood was affected; others took action in order to improve their situation and alleviate the sensations associated with it; and yet others tried to analyze the sensations to find a solution.

*There are sensations that are a little less normal but that are not linked to anything emotional, that require a little more analysis and to see in the long term what, what I can do, is it maybe linked to the contraceptive pill, is it linked to a state that I am not aware of, is it linked to something else. (FG2)*

#### Mind-body connection

One of the themes that emerged from the young adult discourse was the notion of the mind and body as separate yet connected entities, with a tendency to listen more to the body rather than the mind. Two sub-themes emerged from this theme: an understanding of the mind and body as separate and an understanding of the interconnectedness between body sensations, states, and thoughts.

##### Mind-body distinction

The young adult discourse, particularly that of young adult women, showed an understanding of separation between the body and mind. Since the two were perceived as separate, one could choose which one to trust and listen to. There was a general tendency to listen to and trust the body (i.e., bodily signals) rather than the mind (i.e., thoughts).

*It’s only through the body that we have real information, so it’s useful for me to know where I am in my menstrual cycle, in particular so I don’t pay as much attention to the thoughts that I have at certain moments of my menstrual cycle, so typically when I’m approaching my period, when everything is going wrong and it’s the end of the world and I’ll never get to the end of my master thesis, etc. (laughs) I can say to myself: “Oh, okay, I’m having sensations that indicate to me that there’s something talking up there, but we’re not going to believe it,” and this allows me to know what is useful in my life and what isn’t, in terms of what my mind is telling me. (FG2)*

##### Sensations, states, and mind

This sub-theme revealed young adults’ conceptual and experiential understanding of the interconnectedness between body sensations, states (e.g., emotions or physical states like tiredness), and thoughts. Most young adults agreed that body sensations and states (both mental and emotional) were like two sides of the same coin, one could not exist without the other. However, one could choose to pay attention to the different elements of experience (i.e., sensations, emotions, and thoughts) separately. Some young adults discussed that becoming aware of their body sensations often helped them understand the emotional state they were in, whereas others explained that becoming aware of their emotional state instigated them to attend to their body sensations. Yet others found it difficult to experientially make the connection between body sensations and emotions. Finally, one participant also mentioned that emotion-related body sensations could be induced by thinking certain thoughts (e.g., like in gratitude practices).

*I think that the three [body sensations, emotions, and thoughts] are articulated but that one can pay attention to these elements separately… That the three influence each other all the time but that it is possible to focus on one of the three, even if all three are mobilized all the time, in my opinion. (FG4)*

#### (Mal)adaptivity of BA

This theme addresses the question of whether young adults perceive BA as adaptive or maladaptive. Young adults described specific situations in which it was adaptive to be aware of one’s body sensations and situations in which BA was maladaptive or even dysfunctional.

##### Adaptive

Young adults mentioned numerous reasons why BA was adaptive for them. They reported that BA was adaptive for the following: emotion regulation, savoring pleasant sensations, respecting the body’s limits, preventing severe physical problems (e.g., severe back pain) or injuries, understanding one’s emotions, choosing what action to take, anticipating one’s menstrual cycle, choosing what one wants and does not want in life, working with horses, finding a healthy balance, getting to know oneself, and indicating when one is stressed. One participant shared how BA helped her recover from eating-related issues.

*Personally, being aware of my body has helped me a lot to get out of destructive spirals, you know, especially in adolescence, 14–15 years old, in terms of eating habits… so personally I had a phase that was more problematic in that respect and uh we’ll say that I lost a lot of weight intentionally but it was in fact negative in the end and it is after being aware again of my body that told me ah finally stop here or don’t force this thing or at that level you are fine, that I was able to reconcile myself with my body but also mentally that allowed me to have a better balance…. (FG2)*

##### Maladaptive

Young adults engaged in a rich discussion regarding whether BA is maladaptive. Some participants could not think of any situation in which BA was maladaptive. Others, however, gave examples of moments in their lives when paying attention to body sensations led to negative outcomes. Some examples include: when attending to unpleasant body sensations leads to dark ideas and dysphoria (e.g., in the case of a transgender person whose body sensations were a constant reminder of their sex assigned at birth); when BA becomes obsessive and hypervigilant (e.g., in the case of eating-related issues); and when it leads to unconstructive rumination (e.g., constantly asking oneself why one has a certain body sensation). In the young adult women group, the conclusion was that BA itself was not maladaptive but the way one “reads” body sensations and how these sensations relate to the external context determine whether it is adaptive or not.

*I don’t put the word harmful on the bodily sensation as such…and on the fact of paying attention to one’s body, it’s rather uh the reading that we make of it because if we are always blocked there and always trying to analyze it, to understand and all that, and that we go round and round trying to analyze the sensation of which we are conscious, well for me it’s a little bit that which is harmful in fact but not the sensation or the awareness itself. (FG2)*

## Discussion

**Figure 1:**
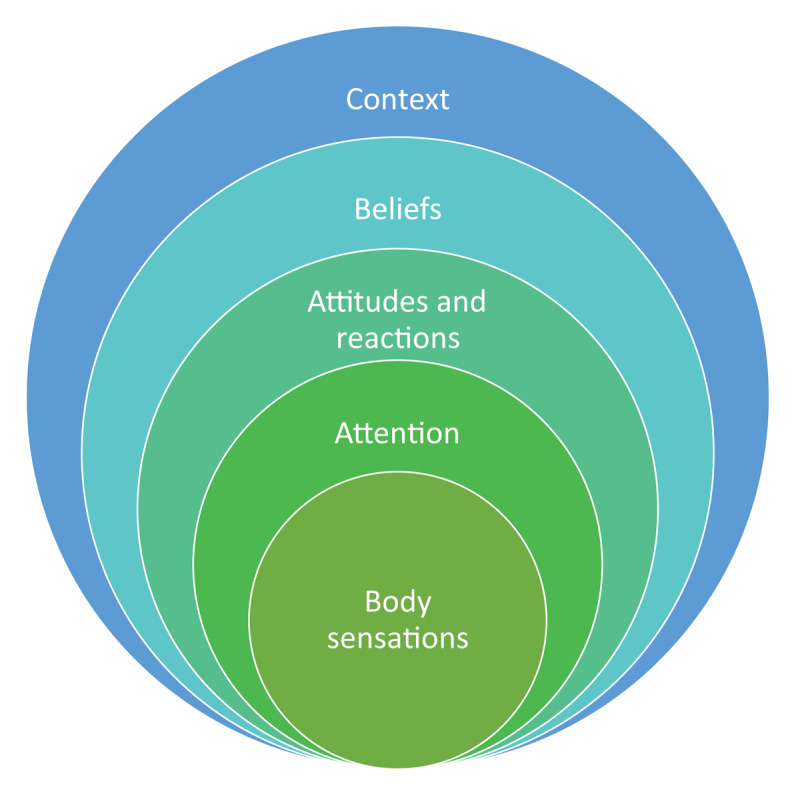
An illustrated summary of youth’s understanding and experience of body awareness as a multifaceted construct.

The purpose of the present study was to explore adolescents’ and young adults’ understanding, experience, and description of BA using a qualitative approach. The analyses of four FGs with adolescents and young adults revealed a multidimensional and highly contextualized understanding and experience of BA across age and gender. Several layers of BA were discussed starting with the content of BA: body sensations that were categorized similarly across groups (i.e., sensations related to emotions or physiology). Then, there was the dimension of attention paid to the content of BA which was contingent upon various factors such as the intensity and valence of body sensations (i.e., intense and unpleasant sensations were noted quicker and more often). Next, were the cognitive, emotional, and behavioral reactions to these sensations that were colored by a particular attitude (e.g., mindful and accepting or avoidant and obsessive). The previous layers were embedded in a larger layer, beliefs about BA. There was, for example, the general belief that awareness of body sensations in itself was adaptive, but the particular combination of the different aspects of BA (e.g., interpretation, attitude, reaction, and context) could render it maladaptive. Finally, there was an overarching layer of physical, temporal, mood-related, social, and cultural contexts which affected all other layers (e.g., body sensations were more intense and attended to when doing certain activities like sports or smoking; an obsessive attention to bodily signals was fostered in a culture that promoted a particular body image). The understanding of BA that emerged from the youth’s discourse is in line with models of BA emphasizing its multifaceted nature (Cioffi, 1991; Farb et al., 2015; Mehling et al., 2012; Suksasilp & Garfinkel, 2022). Figure 1 illustrates a summary of the above explanation.

An important take-away from the present study’s results is that BA is a multidimensional and complex concept in the eyes of this group of young people. As mentioned in the introduction, we set out to explore three dimensions of BA: interoceptive attention, self-report and interoceptive beliefs, and attributions of interoceptive sensations. Our results reveal that not only is the BA concept multifaceted, but so are its dimensions.

Regarding interoceptive attention, results show that the tendency to pay attention is affected by many factors. First, the saliency of the bodily stimulus is important. There is a greater tendency to attend to intense body sensations and ignore non-salient sensations. Second, the external context plays a role in participants’ tendency to attend to their body sensations. A non-salient external context increases the tendency to attend to internal body sensations. For example, attention to the body becomes stronger before sleeping when there are fewer external stimuli competing for attention. Third, past experiences seem to shape young people’s tendency to attend to their bodily signals, with those who had participated in mind-body therapies and those who had experienced medical issues, having a greater tendency to attend to bodily signals, regardless of saliency and context. This suggests that the conscious tendency to attend to bodily signals is malleable and can be trained. These observations are in line with Cioffi (1991)’s reflections on somatic attentional strategies. Finally, young people reported that their tendency to attend to body sensations was affected by their socio-cultural context, adding a relational and cultural layer to interoceptive attention that has been studied a bit in adults but less so in youth (Arnold et al., 2019; Freedman et al., 2021; Ma-Kellams, 2014; Zhou et al., 2021). Regarding relational contexts, participants reported attending to their body sensations less when interacting with friends, which is in line with the claim that social situations reduce BA by shifting attention externally rather than internally (Arnold et al., 2019). Future research could explore the impact of BA on social connection in youth, a period of life in which peer relationships take on a special importance (King et al., 2018; Klimes-Dougan et al., 2014; Silvers, 2022). Regarding cultural context, female participants in the young adult FGs explained the impact that cultural messages about beauty ideals had on their BA. Future research could further assess cultural differences in BA in youth. For instance, would youth from East Asian cultures where BA is more emphasized (Ma-Kellams, 2014), have more tendency to attend to their body sensations than youth in Western cultures?

Results also inform the dimension of self-report and interoceptive beliefs, a broad category including a person’s subjective experiences of and beliefs about body sensations and BA (Suksasilp & Garfinkel, 2022). This dimension has been previously criticized for being ambiguous, overly focused on accuracy (i.e., how accurately people think they perceive body sensations), and not paying enough attention to how (i.e., with what attitude) people pay attention to body sensations (W. Mehling, 2016). The aspect of attitude emerged in all FGs, underlining its importance in youth’s experience and understanding of BA. A variety of attitudes were mentioned (e.g., hypervigilant, mindful, avoidant, trusting, etc.). These attitudes were linked to youth’s beliefs about BA’s adaptivity. There was a general belief that BA is maladaptive when it is characterized by a ruminative and hypervigilant attitude, but that it is adaptive when one pays attention openly and receptively. According to various participants, this open and receptive attitude can provide important information about oneself. These reflections on attitude and adaptivity echo Mehling and colleagues’ conceptual framework for adults aiming to distinguish between adaptive and maladaptive forms of BA in a clinical setting (W. Mehling, 2016; W. E. Mehling et al., 2009, 2012). Empirical research in adults has indeed shown that heightened BA can lead to both negative (e.g., hypochondriasis, somatization, and anxiety disorders; Aronson et al., 2006; Barsky et al., 1988; Barsky & Klerman, 1983; Cioffi, 1991; Olatunji et al., 2007) and positive outcomes (e.g., increased subjective well-being, pain attenuation, and enhanced emotion regulation; Ferentzi et al., 2019; Gard et al., 2012; Hölzel et al., 2011), and a key feature explaining these opposing outcomes may be attentional style or attitude (W. Mehling, 2016).

Regarding the third dimension, attributions of interoceptive sensations (i.e., people’s appraisal of perceived bodily signals; Suksasilp & Garfinkel, 2022), the present study found a diversity of appraisals in youth. These appraisals can be summarized into three categories: body sensations indicating a physiological need or a medical issue, body sensations indicating an emotional state, and a miscellaneous category of body sensations associated with neither of the latter two (referred to as undefined sensations). This appraisal or automatic categorization motivated the response to perceived body sensations. Generally, physiological needs were listened to (e.g., sleeping when sleepy), emotion-related sensations were analyzed, avoided, suppressed, or accepted, and undefined sensations were analyzed and sometimes led to preoccupations. The two categories that youth reported struggling with most were emotion-related sensations and undefined sensations. Difficulties with the appraisal and coping of emotion-related sensations can be understood in the context of developmental literature showing that brain regions involved in higher-order emotion regulation are still developing during adolescence and emerging adulthood making youth more vulnerable to emotion dysregulation (Ahmed et al., 2015; Casey & Caudle, 2013). Results further show that the way young people interpret and respond to perceived body sensations may differ across individuals. For instance, for a transgender participant, certain body sensations were appraised as reminders of their sex assigned at birth, leading to negative thinking and a negative mood. To shed more light on individual differences in attributions of interoceptive sensations, future research could conduct qualitative and quantitative studies in specific groups of youth, such as in LGBTQIA+ youth and in youth with psychological difficulties such as eating and anxiety disorders.

On top of informing the three dimensions of BA mentioned in the introduction, the FG results provided information on potential developmental and gender differences in BA. As the data was not specifically collected or analyzed to assess developmental or gender differences, the differences explained below can be seen as observations that need to be tested in future research.

Regarding developmental tendencies, we observed that young adults exhibited a more nuanced and complex understanding of BA. This is visible in the number of themes that emerged from the young adult discourse versus the adolescent discourse (7 versus 4). Although the overarching themes overlapped between the two groups, they were more developed in the young adult group. Overall, the young adult discourse reflected a greater BA across contexts as well as a greater ability to attribute meaning to these sensations. Greater self-reported BA in young adults versus adolescents may be attributed to different reasons such as developmental changes (Li et al., 2017; May et al., 2014) and greater exposure to BA-enhancing experiences in young adulthood (e.g., BA trainings, body-based psychotherapy, etc.). Little is known about the developmental trajectory of BA during adolescence and the transition to young adulthood. Preliminary research suggests different developmental trajectories for different interoceptive processes. Children (as young as the age of six), adolescents, and adults activate the same brain regions during a heartbeat detection task measuring interoceptive accuracy (Klabunde et al., 2019). However, brain regions associated with the metacognition of interoceptive processes continue to develop with age (Klabunde et al., 2019; Li et al., 2017), which is in line with the present study’s observations. Another potential reason for the observed difference could be that young adults have a richer vocabulary to talk about BA than adolescents which would indicate a difference in verbal capabilities but not necessarily interoceptive abilities. In short, the present research suggests an increased and more contextualized self-reported BA in young adults than in adolescents, in this sample. However, this observation needs to be empirically tested using quantitative approaches with self-report measures but also behavioral and neural measures to control for verbal capabilities.

We also observed gender differences in the understanding, experience, and description of BA. This is evidenced by the fact that five of the sub-themes were only present in the women’s discourse. Furthermore, female participants used more nuanced and differentiated vocabulary to describe their bodily experiences. Based on the themes, female participants displayed a greater awareness of the kinds of sensations they paid attention to (e.g., not simply intense but also novel and problematic), a more trusting attitude toward their body sensations; a greater history with medical and emotional issues and body therapies that helped, and a greater awareness of the connection and distinction between the mind and the body. Hence, in this sample, female participants reflected higher BA. These findings are in line with research on gender differences in interoception in adults which shows that women have higher interoceptive sensibility than men (Longarzo et al., 2021), particularly when it comes to noticing body sensations and linking them to emotions (Grabauskaitė et al., 2017). These gender differences have been theorized to derive from the fact that women experience more hormonal and physical changes throughout their lives than men (e.g., menstruation, pregnancy, menopause) making them more attentive to internal bodily signals (Murphy et al., 2019). The FG results support this theory as young women spontaneously discussed menstruation as an important time in which they paid attention to their bodily signals. Hence, the present study’s observations are in line with previous findings on gender differences in interoception in adults. However, this observation that female participants exhibited higher BA than male participants must be considered in light of the fact that the female young adult sample was older and more highly educated than their male counterpart. Moreover, all co-facilitators were women which may have favored self-expression and disclosure in the female versus the male groups. Finally, there were more female than male participants in the study. Hence, future studies should test gender differences in BA in a representative population of youth containing samples of youth matched in age and education and including male facilitators for male FGs.

Finally, the researchers made observations on the way young people spoke about BA. The language used was simple and concrete. Words such as “body sensations” were not immediately understood by all participants, so the facilitator needed to provide explanations and concrete examples. Often terms like “emotions” and “body sensations” were used interchangeably. These observations on language may have implications for future research described next.

Overall, our results show that the phenomenological experience and understanding of BA in youth is multidimensional, concrete, may be increasingly nuanced with age, and may be influenced by gender. This has implications for future theoretical frameworks and self-report measures for youth. The first implication is that it is important for future theoretical frameworks and self-report measures to be multidimensional to capture the complexity of the BA construct. Three important dimensions that arose from the FGs with youth were: the tendency to pay attention to body sensations and the many factors influencing this, the attitude with which one pays attention and its importance in determining whether BA is adaptive or not, and the diversity of reactions to body sensations depending on how they are appraised. A second implication is that since there may be gender and developmental differences in BA, which need to be confirmed in future studies, future and existing BA self-report measures for youth should be tested for measurement invariance across age and gender. A third implication is that since youth may lack the vocabulary to talk about BA, future self-report measures must use concrete and simple language, with short explanations for psychological terms such as “emotion” and “body sensations”. By using theoretical frameworks and self-report measures tailored for youth, research will be able to better understand the developmental trajectories of BA in youth. This in turn could paint a clearer picture of the role of BA in the development, treatment, and prevention of psychopathology in youth.

These results must be considered in light of the present study’s limitations. First, the FGs were done online, so important non-verbal information such as body language was lost; and communication amongst FG participants was sometimes affected by technological difficulties. Moreover, though the group setting is one of the assets of FGs, as it stimulates a rich discussion, it also represents a limitation as it increases potential for social desirability bias and may hinder participants’ full expression of their BA-related experiences and beliefs (Smithson, 2000). A second limitation is that the FG facilitators were all women, which may have favored open expression in the female groups but hindered free expression in the male groups. Third, inherent to the qualitative nature of the study, results are not generalizable, and the sample is not representative of diverse groups and gender identities. Future research could explore similar questions in youth of other ethnicities, nationalities, socio-economic status, and gender identities. Furthermore, quantitative research approaches could be used to address the issues of generalizability and sample representation. A fourth limitation is that the present study did not control for the presence of diagnosed mental disorders, limiting the transferability of results to clinical populations. Furthermore, even though participants were recruited from the general population, there could have been participants with a diagnosed mental disorder in the FGs. This could have had an impact on the thematic results as individuals with mental disorders may report higher levels of unpleasant and intense body sensations accompanied by a negative appraisal of these sensations. Future qualitative studies on BA in youth should control for the presence of diagnosed mental disorders. A final limitation is that the young adult male and female FGs were not well-matched with regards to education level, as 71% of young adult women were doing master studies whereas all young adult men were doing bachelor studies.

Future directions for the current line of research include using the findings that emerged from the present study to inform interoceptive theoretical frameworks for youth and to develop a self-report instrument to measure BA in youth. This will further the understanding of the development of BA in this age group, the link between BA and mental illness, and the role of BA in psychological interventions in youth.

## Data Accessibility Statement

Some of the data that support the findings have been included in the manuscript and in the supplementary materials. The rest of the data are available upon request from the corresponding authors, MPP and PP. The data are not publicly available due to restrictions i.e. their containing information that could compromise the privacy of research participants.

## Additional File

The additional file for this article can be found as follows:

10.5334/pb.1295.s1 Supplementary Materials. Additional information on participants’ demographic characteristics as well as additional quotes for each theme.
