# Supplementary material for: Body Aware: Adolescents’ and Young Adults’ Lived Experiences of Body Awareness
Source: Psychol Belg. 2024 Aug 12;64(1):108–28. doi: 10.5334/pb.1295 (PMC11328682; doi:10.5334/pb.1295)
Supplement: Supplementary Materials. — Additional information on participants’ demographic characteristics as well as additional quotes for each theme. [file pb-64-1-1295-s1.pdf]

Supplementary Materials

S1 provides additional information on the demographic characteristics of participants per focus group. This may help the reader further contextualize the findings.

S2 provides additional quotes organized per age group and per theme. Since only a few quotes were included in the results section, these additional quotes can provide more information that may help the reader refine their understanding of specific themes.

S1: Participant characteristics

**FG with female adolescents (FG1)**	(*N* = 4)
Age (*M*, *SD*)	15.75 (0.5)
Female gender	4
Year of studies (secondary school)	
9th grade	1
10th grade	1
11th grade	2
Type of studies	
General	4
Belgian nationality	4
French as mother tongue	4
**FG with female young adults (FG2)**	(*N* = 7)
Age (*M*, *SD*)	22.29 (1.98)
Female gender	7
Year of studies (university)	
Bachelor	2
Master	5
Type of studies	
Psychology	3
Communication	2
Audiovisual	1
Speech therapy	1
Nationality	
Belgian	6
French	1
French as mother tongue	7
**FG with male adolescents (FG3)**	(*N* = 5)
Age (*M*, *SD*)	15.6 (0.55)
Male gender	5
Year of studies (secondary school)	
11th grade	2
9th grade	2
Unknown	1
Orientation of studies	
General	3
Technical	1
Unknown	1
Belgian nationality	5
French as mother tongue	5
**FG with male young adults (FG4)**	(*N* = 4)
Age (*M*, *SD*)	20.25 (1.71)
Gender	

Male	3
Other	1
Year of studies (university)	
Bachelor	4
Type of studies	
Psychology	1
Marketing	1
Social work	1
Biomedical sciences	1
Belgian nationality	4
French as mother tongue	4

S2: Supplementary focus group quotes organized by group and themes

Adolescent results

Attention

Attention grabbers

- So I when I really observe the sensation is when it's really too strong and it really takes over me so I try to stop myself and understand what is happening… (FG1)
- Participant: But if not, I notice unusual things more often.
  Facilitator: And what are unusual things for you?
  Participant: Well becoming really angry, I don't usually get angry so…I don't really know… or when I experience a big stress but that also doesn't happen every day… (FG1)
- Yes, if not, for me it's especially tiredness, it's a bit like Participant 25 but I pay attention to tiredness even if it's sometimes a bit in my daily life because sometimes it prevents me from doing things in my daily life… (FG1)

Temporality of attention

- I would say both [paying attention during and after perception of sensations] because I sometimes have some discomfort in my belly but I don't really pay attention and then maybe afterwards, a few minutes after really having a stomach ache and then and then I tell myself in that moment yes, it's true, ten minutes ago I also already had that but I was just not paying attention because I was busy doing something else… (FG3)

Awareness of body sensations

Types of sensations

- When I feel angry I sometimes feel, I feel something in my arms, I feel like breaking everything, I feel something itchy in my arms. (FG3)
- When I'm hungry, I have gurgling pains and all that. I feel a sense of urgency to eat. (FG1)
- It's fatigue, a sensation in which your eyes often sting, I don't want to do anything anymore. (FG3)

Lack of awareness

- I don't really pay attention to what I feel in my body, except when I'm hungry and when I'm tired. (FG3)

Reactions to body sensations

Hedonic

- Participant: And uh when I feel a positive sensation, well I try to... to make it last.
  Facilitator: And what do you do to make it last?
  Participant: Well, I continue to do what gives me that feeling, I don't know... for example I'm laughing with a friend and I'll try to continue to have a good time with her and when she leaves or whatever I'll try to remember the moments we had and generally it makes me smile… (FG1)

Listening

- But now that we're in lockdown, well when I get up I don't force myself to eat, I wait a little until I'm really hungry and tell myself what am I going to eat? (FG1)

Instrumental

- When we do sports and well the body sends signals like we are suffering and so we must not listen to it otherwise we give up, we don't evolve and there you go. (FG3)

Beliefs about BA

Adaptivity

- Yes, it's useful because I think that the body sends us signals and that it's a bit like an alarm signal to say ah uh your body tells you that you're hungry uh that you're sleepy and so you have to know how to listen to it. (FG3)

Consequences of not listening

- "We must not not pay attention to it [body sensations] because it can become harmful, for example, if we don't pay attention to the fact that we are tired, the next morning we have less energy and all that." (FG3)

Good balance

- I think we have to pay attention, but we shouldn't let it eat us up either, we shouldn't think about it day and night, saying to ourselves ah why did it hurt that day? Why did I feel sad? Why did I have a lump?... (FG1)

Link between states and sensations

- Well, sometimes I... I don't know... yes I have I have sensations but we'll say unexplained that come or whatever…uh for example I slept well or things like that but uh I have a feeling of heaviness like when I'm tired or things like that but uh so I don't really consider that it's fatigue but uh I don't know sometimes it happens to me but it's still very rare in general it's related. (FG1)

Young adult results

<u>Attention</u>

Types of attention

- I realize that I don't pay any attention to my sensations at all, not at all, but when they come and I feel them, well, of course they come, I don't pay special attention to them but I feel them. (FG4)

<u>Body sensations</u>

Types

- There are sensations that are a little less normal but that are not linked to anything emotional, that require a little more analysis and to see in the long term what, what I can do, is it maybe linked to the contraceptive pill, is it linked to a state that I am not aware of, is it linked to something else. (FG2)

Impermanent

- When it's uh uh a feeling due to uh a rather important irritation or uh or a deep sadness for example uh then there uh uh I can only count on time so that it attenuates and returns to a normal state. (FG4)

<u>Processes</u>

Interpretation

- … for me it's not what you feel that is the problem, it's how you interpret it... (FG2)

Attitudes

- Sometimes if something happens in our body, we will want to analyze it at all costs and so on and we will want to know exactly what it is and this can turn into an obsession or we can misinterpret it or something like that and I think that it can be harmful because it makes us ruminate, there are always the same ideas coming back because it's like oh I feel that, but why can't I find the cause? And I feel that way and so in the end it makes me ruminate and it annoys me more than anything else in fact and so it's a bit harmful because it goes round and round in a loop, uh it doesn't change anything, yes. (FG2)

- …almost every hour in my opinion there is at least one time when I say to myself uh where am I? Uh what is it? You come back to your body or I come back to the present moment through my senses or I try to cut off the mind. For example, so even more so in this period, when I pet my cats I try to really feel what's going on, same when uh I go to see my horses uh every time I go to see them I come back to my feet, I feel my feet, I feel when I'm walking near them, I feel my breathing, I listen to the sounds around uh so I pay attention to the sensations in my body too. So, when I do this process of coming back into my body I do with what's there or I connect to the parts of the body that are easier to reach so I feel what's going on under my fingers or in my feet. (FG2)

Reactions to body sensations

Reactions to physical sensations

- …there are sensations that I consider more physiological such as I am thirsty, I am hungry, uh I am tired, well at that moment uh it depends on the moment but most of the time I try to just uh meet this need linked to the sensation, for example if I'm really thirsty I'll go and get a glass of water, if I'm hungry I'll maybe try to open the fridge to see if there's something I could eat so it's nothing special. (FG4)

Reactions to emotion-related sensations

- If I notice a sensation that was linked to a strong emotion of sadness or something like that, well I realize that it's negative so at that moment I say to myself uh that anyway it's ruining what I'm doing at the moment and so one of the only ways to deal with it if I can't do anything else, if I can't go directly to the source, then I say to myself, too bad, I give up what I'm doing for the moment, I cry a little bit because I have the right to feel what I feel and then I let myself go a little bit because I say to myself, at the end of the day, we have the right to feel these feelings and that's it. (FG2)

Reactions to undefined sensations

- The sensation uh uh hm (laughs) how to explain that... for example uh let's take the case of a transgender person FTM [female-to-male] for example, uh the sensation of his chest uh compressed by a binder or or something like that uh if he can't uh obstruct this sensation uh it's a constant reminder uh to his birth sex, which can uh well then uh uh engender uh dark thoughts and a uh yes a bad mood and uh etcetera. (FG4)

Mind-body connection

Sensations, states, and mind

- I know that I had a lot of sensations before where I didn't know how to associate them with my emotions, I didn't know what it meant. I felt things and I didn't really know what it meant and uh I didn't know how to react to it and... now that I've associated certain sensations to feelings, it's much clearer how I function, I know myself much better and uh analyzing these sensations well it allowed me to understand myself much better and to get to know myself too. (FG2)

<u>(Mal)adaptivity</u>

Adaptive

- Personally, being aware of my body has helped me a lot to get out of destructive spirals, you know, especially in adolescence, 14-15 years old, in terms of eating habits, it is sometimes problematic, so personally I had a phase that was more problematic in that respect and uh we'll say that I lost a lot of weight intentionally but it was in fact negative in the end and it is after being aware again of my body that told me ah finally stop here or don't force this thing or at that level you are fine, that I was able to reconcile myself with my body but also mentally that allowed me to have a better balance, so it allowed me to have a healthier life both physically and mentally and by paying attention to these body signals, that finally the weight at which I felt good was not necessarily the weight that was the lowest, but the moment when I felt the best in my body was a little bit in another place, or by doing perhaps this type of exercises rather than others, by eating such things rather than others. And so, it really allows you to pay attention to what your body is saying and it allows you to be more balanced on all levels. (FG2)
